# Urokinase-Type Plasminogen Activator Receptor Regulates Prosurvival and Angiogenic Properties of Cardiac Mesenchymal Stromal Cells

**DOI:** 10.3390/ijms242115554

**Published:** 2023-10-25

**Authors:** Konstantin Dergilev, Zoya Tsokolaeva, Yulia Goltseva, Irina Beloglazova, Elizaveta Ratner, Yelena Parfyonova

**Affiliations:** 1 Institute of Experimental Cardiology Named after Academician V.N. Smirnov, Federal State Budgetary Institution National Medical Research Center of Cardiology Named after Academician E.I. Chazov, Ministry of Health of the Russian Federation, 121552 Moscow, Russia; kvdergilev@cardio.ru (K.D.);; 2 Federal Research and Clinical Center of Intensive Care Medicine and Rehabilitology, 107031 Moscow, Russia; 3 Department of Biochemistry and Molecular Medicine, Faculty of Medicine, Lomonosov Moscow State University, 119192 Moscow, Russia

**Keywords:** cardiac mesenchymal cell, CD117, uPAR, uPA, cell survival, angiogenesis, cardiac repair

## Abstract

One of the largest challenges to the implementation of cardiac cell therapy is identifying selective reparative targets to enhance stem/progenitor cell therapeutic efficacy. In this work, we hypothesized that such a target could be an urokinase-type plasminogen activator receptor (uPAR)—a glycosyl-phosphatidyl-inositol-anchored membrane protein, interacting with urokinase. uPAR is able to form complexes with various transmembrane proteins such as integrins, activating intracellular signaling pathway and thus regulating multiple cell functions. We focused on studying the CD117+ population of cardiac mesenchymal progenitor cells (MPCs), expressing uPAR on their surface. It was found that the number of CD117+ MPCs in the heart of the uPAR−/− mice is lower, as well as their ability to proliferate in vitro compared with cells from wild-type animals. Knockdown of uPAR in CD117+ MPCs of wild-type animals was accompanied by a decrease in survival rate and Akt signaling pathway activity and by an increase in the level of caspase activity in these cells. That suggests the role of uPAR in supporting cell survival. After intramyocardial transplantation of uPAR(−) MPCs, reduced cell retention and angiogenesis stimulation were observed in mice with myocardial infarction model compared to uPAR(+) cells transplantation. Taken together, the present results appear to prove a novel mechanism of uPAR action in maintaining the survival and angiogenic properties of CD117+ MPCs. These results emphasize the importance of the uPAR as a potential pharmacological target for the regulation of reparative properties of myocardial mesenchymal progenitor cells.

## 1. Introduction

All adult mammalian organs, including the heart, contain resident stem/progenitor cells that form a specific microenvironment and are responsible for maintaining tissue homeostasis. Mesenchymal stromal cells (MSC) are considered to be a key component of this microenvironment and represent heterogeneous cells of different types. In recent years, attention has been attracted to the study of tissue-specific populations of MSC and the mechanisms of their regulation. One of such MSC populations is cardiac mesenchymal progenitor cells (MPC), which are localized in perivascular or/and intramyocardial niches and involve in cardiac repair [1]. These cells can be isolated from human and animal heart tissue and possess the defining stem/progenitor cell characteristics, being self-renewing, clonogenic, and multipotent and differentiating into adipocytes, osteocytes, and cardiac cell types in vitro and in vivo [2,3,4,5,6,7,8,9,10,11]. The ability of MPCs to support cardiac repair via the generation and secretion of prosurvival and reparative growth factors is widely recognized [2,12,13]. These properties of MPCs make them an attractive tool for cell therapy of cardiac diseases due to cardioprotective effects, stimulation of angiogenesis, modulation of immune reactions, inhibition of fibrosis, and proliferation of resident cardiac progenitor cells. Despite promising results and evidence of safety in preclinical studies using MSCs, the effects reported in clinical trials are not conclusive and even contradictory [14]. One of the reasons may be the lack of specific cell surface markers; the variability in the number of obtained cells and their functional properties indicates a high degree of population heterogeneity [15,16], which may reflect the diversity of microenvironment present in the natural niches of stem cells. The heterogeneity of MSCs may reduce their therapeutic efficacy and introduce differences between studies [15]. The use of specific MSC subpopulations may increase the homogeneity of the cellular product and specificity for a certain type of pathology and develop more effective therapies. Therefore, it is important to identify specific markers of MSC populations, subfractionate them, characterize their reparative properties to standardize therapy, and develop personalized treatments for heart disease [17].

Among the many well-known regulators of cell function and repair processes in the heart, the multifunctional urokinase (uPA)/receptor urokinase (uPAR) system deserves certain attention [18,19]. Initially, the glycosylphosphatidylinositol (GPI)-anchored membrane protein uPAR was considered exclusively for its ligand—urokinase, the interaction with which caused the conversion of plasminogen to plasmin and thereby the regulation of cell surface proteolysis [20]. However, it was later found that independent of proteolytic function, uPAR modulates cell proliferation, cell adhesion, and migration of various cell types through interaction with signal transducers and the activation of intracellular signaling pathways [21,22,23,24,25]. The involvement of the uPA/uPAR system in the regulation of cardiac MPC properties and functions remains, however, unexplored. Therefore, our study was designed to investigate the role of the uPA/uPAR system in the regulation of CD117+(c-kit+) subpopulation of cardiac MPCs functions. We provide evidence that uPAR is required for CD117+ MPCs survival/proliferation, post-transplantation retention, and reparative properties in vivo.

## 2. Results

### 2.1. CD117+ MPCs Express an uPAR Receptor

**Figure 1:**
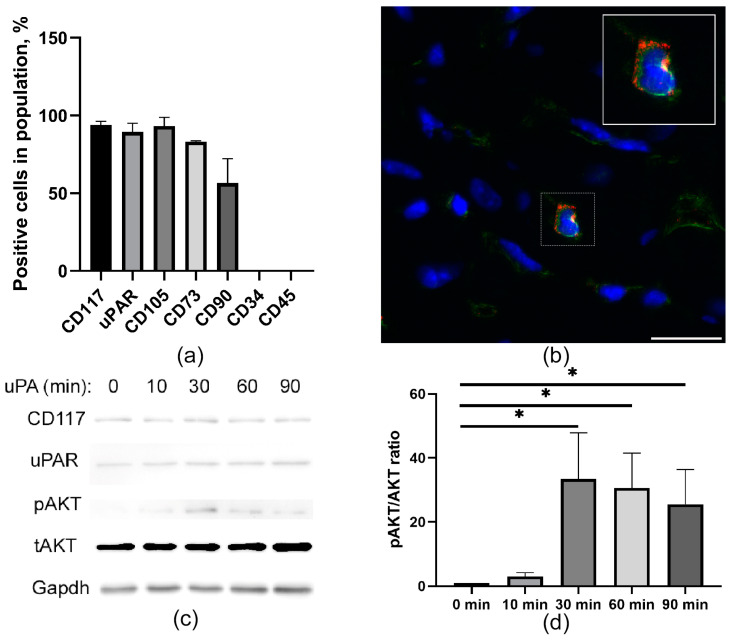
Cardiac MPCs characterized by the expression of uPAR. (**a**) Flow cytometry analysis of expression of CD117, uPAR, mesenchymal (CD105, CD73, and CD90), and hematopoietic markers (CD34 and CD45) in MPCs. The data on the graph are presented as mean ± SD. (**b**) Representative image of CD117 (red) and uPAR (green) marker expression in MPCs located in the myocardium. Scale bar 20 mkm. (**c**) Time course of AKT activation. Cells were serum-starved and incubated with uPA (10 nM) in serum-free medium at different time points (minutes): 0, 10, 30, 60, and 90 min. The cells were lysed in RIPA buffer and immunoblotted with anti-AKT, anti-pAKT, CD117, uPAR, and Gapdh antibodies, respectively. Shown in the bar graph (**d**) are the mean (±SEM) of three experiments expressed as pAkt/Akt ratio, normalized to the levels of unstimulated cells that were assigned a value of 1. * *p* < 0.05.

CD117+ MPCs were characterized by the expression of uPAR (93.9 + 2.3%) and display of mesenchymal stem cell markers CD105, CD73, and CD90, which is combined with a lack of CD45 and CD34 on the cell surface (Figure 1a,b). We tested the functional activity of the uPAR associated with triggering intracellular signaling using recombinant uPA. We found that AKT phosphorylation in CD117+ MPCs after uPA treatment peaks after 30 min and drops gradually by 90 min (Figure 1c,d). These data suggest that CD117+ MPCs contain a functional uPAR receptor that enables the uPA-induced AKT signaling pathway.

### 2.2. uPAR−/− Mice Are Characterized by a Reduced Number of CD117+ MPCs in the Heart, Compared to Wild-Type (uPAR+/+) Animals

**Figure 2:**
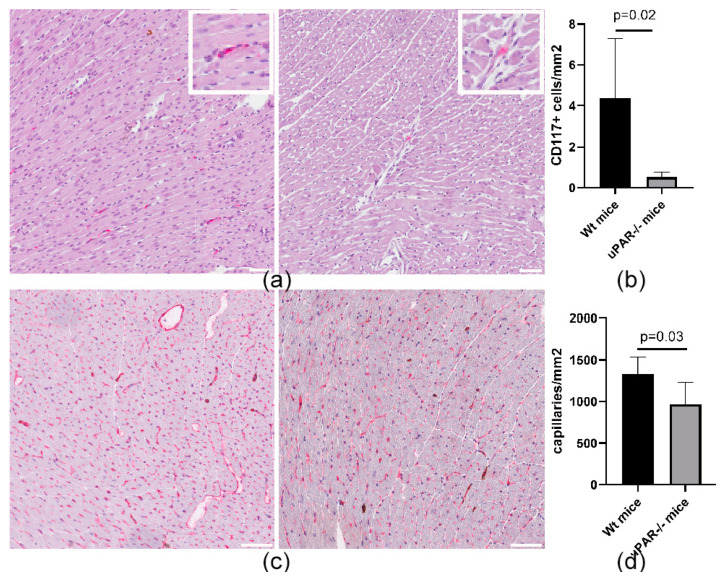
uPAR−/− mouse hearts are characterized by reduced CD117+ MPCs population and signs of vasculopathy. (**a**) CD117+ MPCs are localized in the hearts of Wt (left) and uPAR−/− mice (right). Heart slices were immunohistochemically labelled with anti-CD117 antibody (red). Cell nuclei were stained with hematoxylin. (**b**) CD117+ MPCs count in the hearts of Wt and uPAR−/− mice (four animals in each group). Data are presented as mean ± SD. (**c**) Representative images of double immunohistochemical staining of cardiac vessels (CD31 (red) and SMA (brown)) in the hearts of Wt (left) and uPAR−/− (right) mice. Cell nuclei were stained with hematoxylin. (**d**) capillaries count in the hearts of Wt (left; *n* = 4) and uPAR−/− (right; *n* = 6) mice. Data are presented as mean ± SD. Scale bar 150 mkm.

Taking into consideration that the PI3K-AKT signaling pathway plays an important role in promoting survival and apoptosis in a wide range of cell types, we evaluated the content of CD117+ MPCs in the hearts of wild-type and uPAR−/− knockout mice. The number of MPCs in the heart of the uPAR−/− mice was eight times lower than that in wild-type animals (Figure 2a,b), which was combined with a decrease in myocardial capillarization, compared to wild-type animals (Figure 2c,d).

### 2.3. Knockdown of uPAR Is Accompanied by a Decrease in Prosurvival Properties in MPCs

**Figure 3:**
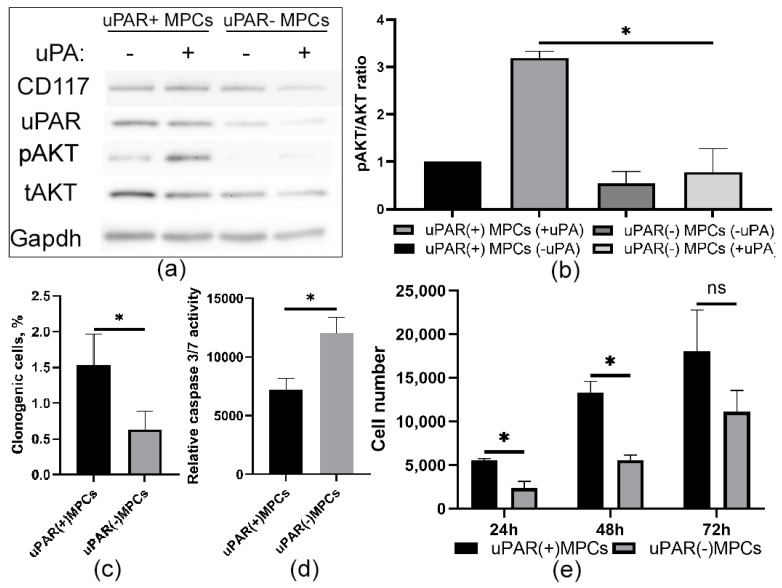
Knockdown of uPAR decreases uPA-mediated Akt signaling and prosurvival properties of MPCs. (**a**) The knockdown efficiency of shRNA transduced by lentivirus, targeting uPAR in MPCs determined by Western blot. uPAR(+) and uPAR(−) MPCs were serum-starved and incubated with uPA (10 nM) in a serum-free medium for 30 min. The cells were lysed in RIPA buffer and immunoblotted with anti-uPAR, AKT, pAKT, CD117, and Gapdh antibodies, respectively. Shown in the bar graph (**b**) is the mean (±SEM) of three experiments expressed as pAkt/Akt ratio. * *p* < 0.05. (**c**–**e**) Suppressed prosurvival properties after uPAR downregulation were observed in MPCs by clonogenic assay (**c**), caspase 3/7 activity analyses (**d**), and MTT assay (**e**). Results are presented as average values obtained in at least three repeats.

To explore the potential role of uPAR in cell survival, we stably transfected either mice uPAR-targeted shRNAs or scrambled shRNA as a control via lentiviral infection. Western blot analyses showed that viral transduction suppressed uPAR expression level (Figure 3a) by more than half, reduced clonogenic properties (Figure 3a), and prevented AKT phosphorylation after stimulation with recombinant urokinase, compared to control MPCs (Figure 3a,b). Furthermore, in CD117+ MPCs, stable uPAR downregulation significantly suppressed proliferation, as determined by the MTT test (Figure 3e). Furthermore, the presence of the uPAR also prevents apoptosis induced by serum depletion in MPCs. For this purpose, we measured the activation of caspases 3 and 7 in MPCs for 72 h. As shown in Figure 3, the loss of uPAR resulted in a significant increase in caspase activity in these cells compared to control uPAR+ MPCs (Figure 3d). Taken together, these results show that the presence of uPAR in MPCs is involved in the realization of prosurvival effects.

### 2.4. uPAR Knockdown Reduces MPCs Posttransplantation Retention and Decreases Reparative Angiogenesis Activity after Myocardial Infarction

**Figure 4:**
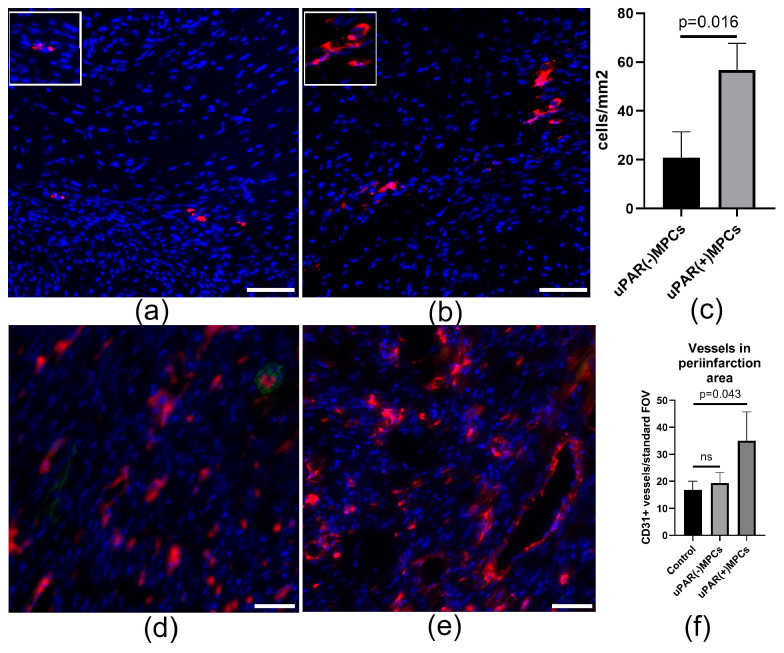
Downregulation of uPAR reduced posttransplantation MPCs retention and vascularization after myocardial infarction. (**a**,**b**) Representative images of uPAR(−) (**a**) and uPAR(+) MPCs (**b**), labelled with the red fluorescent dye CM-DIL, in mice myocardium 72 h after transplantation. (**c**) Graphs quantifying the number of uPAR(+) and uPAR(−) MPCs retained in the myocardium 72 h after transplantation (*n* = 4). (**d**,**e**) Representative images of vascularization of the periinfarction region of the hearts after transplantation of uPAR(−) (**d**) and uPAR(+) MPCs (**e**). Vessels were stained with antibodies against endothelial marker CD31 (red) and cell nuclei—with Dapi (blue). (**f**) Graphs quantifying the total number of CD31+ vessels in periinfarction zones (7 days after MI) after transplantation of uPAR(+) and uPAR(−) MPCs (*n* = 4). Scale bar 100 mkm.

To examine whether uPAR receptor modulation in MPCs can influence repair processes after cardiac injury, we used an animal model of myocardial infarction. Intramyocardial transplantation of fluorescently labelled MPCs after myocardial infarction was performed. uPAR(+) and uPAR(−) MPCs were stained with lipophilic cell membrane dye (CM-DiI) and injected imtramyocardially in the border zone 1 h after the surgery. Fluorescently labelled uPAR(+) MPCs were detected 72 h after transplantation at the site of injections. The opposing situation was observed with uPAR(−) MPCs, injected cells predominantly failed to engraft the injury tissue (Figure 4a–c). In both cases, however, we did not observe signal co-localization of the CM-Dil and CD31 markers, indicating that the transplanted cells do not show signs of differentiation in the endothelial direction. Meanwhile, uPAR(+) MPCs transplantation significantly increased the number of vessels in the periinfarction area compared to the uPAR(−) cell injection group (Figure 4d–f), which may indicate the angiogenic properties of their secretome.

### 2.5. uPAR−/− MPCs Showed Reduced Proangiogenic Secretory Activity and Limited Ability to Stimulate Angiogenesis In Vivo

**Figure 5:**
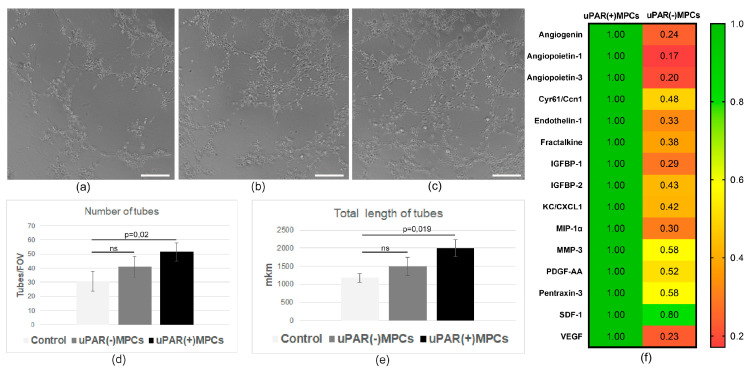
Knockdown of uPAR reduces secretion of proangiogenic factors and angiogenesis properties in vitro. (**a**) Representative images of tube-like structures on Matrigel (angiogenesis assay), formed by mouse endothelial cells in control medium (**a**) and after treatment with conditioned mediums of uPAR(−) (**b**) and uPAR(+) MPCs (**c**). (**d**,**e**) Graphs quantifying the total number and length of tube-like structures (*n* = 3). Scale bar 200 mkm. (**f**) Heatmap of uPAR(+) and uPAR(−) MPCs secretome analyzed by Proteome Profiler Mouse Angiogenesis Array Kit.

On the basis of the findings indicating a lack of differentiation of the transplanted cells in the endothelial direction, we investigated the effect of the secretome on angiogenesis in vitro. Exposure of the endothelium with uPAR(−) MPC’s secretome resulted in a 20% decrease in the number of vascular structures, with an average length one-quarter that of the uPAR(+) cells secretome (Figure 5a,b). In addition, the uPAR(−) cells secreted less proangiogenic factors (VEGF, angiopoietin 1, angiopoietin 3, endothelin 1, and others) in comparison with uPAR(+) MPCs (Figure 5c), which indicates an altered proangiogenic paracrine function.

## 3. Discussion

Cell therapy based on the use of CD117+ MPCs opens up opportunities for the treatment of heart diseases [26]. Studies on both animal models and humans have shown the safety and promising results of this treatment [27]. Meanwhile, many questions remain regarding the principles of regulation of this cell type, methods of cell preparation, and subsequent integration into the myocardium after transplantation, which are directly correlated with the therapeutic efficacy of these cells. A better understanding of the mechanisms regulating CD117+ MPCs functioning is necessary to overcome the limitations of their unique reparative activity after transplantation.

In the present study, we investigated the role of uPAR in the regulation of cardiac CD117+ MPCs. Our data demonstrate for the first time that (1) CD117 + MPCs express the urokinase receptor on their surface; (2) uPAR-deficient animals are characterized by a reduced number of CD117 + MPCs in the heart; (3) the presence of uPAR is important for maintaining proliferation and survival of CD117+ MPCs and is associated with activation of AKT signaling pathway; (4) the presence of uPAR is important for integration of transplanted MPCs into myocardium and stimulation of reparative angiogenesis.

CD117+ MSCs are thought to be a population of quiescent stem cells, which reside in the niche of the heart [28,29]. After myocardial infarction, CD117+ MPCs migrate to the area bordering the infarction through the activation of HGF-c-met, SDF1-CXCR4, and IGF1-IGFR signaling mechanisms [24,25,26,27,28,29,30,31,32,33,34,35], thus providing protection and repair of damaged myocardium presumably by secretion of bioactive compounds [36,37,38,39]. We suggested that the presence of the urokinase receptor on the surface may serve as a signal transducer to trigger the proliferation of MPCs, which is a key step for their activation after myocardial infarction. Currently, available information concerning the urokinase system’s involvement in the regulation of cardiac MPC functioning and mechanisms of cardiac repair is limited. Meanwhile, a significant increase in uPA and uPAR expression patterns at both transcriptional and protein levels was shown by the Stavropoulou study [40]. Furthermore, uPA is involved in the reparative response and the pathogenesis of heart failure development, which indicates an important role of this system in postinfarction recovery [41,42,43].

According to our findings, uPAR−/− animals show a decrease in the number of MPCs as well as a presence of vasculopathy in their hearts. In addition, cell retention in the myocardium after transplantation was significantly reduced in receptor deficiency. Our data have similarities with the principles of stem cell niche regulation in the bone marrow. For these cells, the presence of uPAR is an important condition for the maintenance of hematopoietic stem cells in the BM niche, functioning as an autonomous signal to keep “dormant” cells in the microenvironment. The loss of uPAR associated with cleavage/conversion to soluble uPAR fragments (suPAR) promotes HSC mobilization. Along with the impaired interaction of uPAR−/− cells with niche components, the second cause of the decrease in their number can be the impairment of their proliferative activity. We found that receptor downregulation resulted in a decrease in their proliferation. The reason for that can be a decrease in Akt phosphorylation that we observed upon uPAR downregulation as well. Since uPAR lacks transmembrane or intracellular domains, it needs to interact with transmembrane receptors and complexes to trigger downstream signaling and promote cell proliferation/survival [39]. Interaction of uPAR has been demonstrated with the majority of integrins, with the highest affinity for α3β1 and α5β3, which influences cytoskeleton organization, determines organization and stability of cell adhesion, and regulates migration and proliferation [44,45,46,47]. This is realized through the activation of intracellular signaling pathways that are largely specific to each type of integrin and can include the activation of focal contact kinase (FAK), Src kinase, phosphatidylinositol 3-kinase (PI3K)/protein kinase B (AKT), MAPK kinases (Ras/MAPK/ERK pathway), GTPase Rac, JAK/STAT, and others [46,48]. These findings are aligned with other studies, which have strongly suggested that uPAR downregulation leads to the disruption of intracellular signaling and suppression of cell proliferation [49,50]. In addition, the loss of the receptor can disrupt the DNA repair process [51], which may be one of the causes of the reduced survival/integration rate of uPAR(−) MPCs cells after transplantation.

Thus, we found that the presence of uPAR on the MPC surface is important to provide uPA-mediated activation of the AKT pathway responsible for essential cellular properties. This signaling mechanism is crucial for the regulation of MPC migration, efficient engraftment after transplantation, and performing their reparative properties [52]. A number of researchers argue that the activation of the AKT pathway can also increase the secretion of VEGF and other factors. Numerous inhibitors targeting the PI3K/AKT/mTOR pathway have been developed, and these agents have been shown to decrease VEGF secretion and angiogenesis. This agreed well with the data showing decreased expression of proangiogenic factors in uPAR−/− cells and suppressed angiogenic secretome activity in vitro and in vivo. Moreover, previously published studies have shown that matrix metalloproteinase (MMP)-12 overexpression cleaves uPAR, impairs angiogenic endothelial cell turnover, and inhibits uPA-mediated angiogenesis thus causing vasculopathy [53,54,55,56,57]. This can be explained by the ability of uPA to protect ECs from apoptosis [58,59] and to promote EC proliferation [60]. Moreover, disruption of the interaction uPA with uPAR alters EC functions, including adhesion, migration, proliferation, and capillary tube formation, and reduces EC angiogenic functions [61]. Consequently, it can be assumed that providing uPA/uPAR interaction is important for both MPCs and endothelial cells, which allow regulating the interaction with integrins, VEGFR2, which mediates the formation of vascular structures and ensures effective angiogenesis in the heart [57,62,63,64].

Alternatively, the specialized effects of uPAR+ cells on target cells can be realized by secreting extracellular vesicles, which are lipid nanoparticles secreted by cells that can carry various macromolecules, including proteins and non-coding RNAs. Vesicles can carry biological cargo both locally and remotely through the bloodstream, as well as transfer their contents by cells within the microenvironment to modify their behavior [65]. Several studies have shown that EVs can carry uPAR and modify endothelial cell behavior. Thus, (uPAR)-positive EVs can regulate angiogenesis through overexpression of VE-Cadherin, EGFR, and uPAR and activation of ERK1/2 signaling in endothelial cells [66]. It can be assumed that due to a similar mechanism, uPAR+ EVs can realize the immunoregulatory potential of MSCs. By interacting with specific macrophage receptors (Mac-1 (CD11b/CD18) and fMLF receptors), uPAR can participate in the regulation of migration, differentiation, and phagocytosis modulating the level of inflammation in the heart tissue [67,68,69,70,71]. Consequently, the use of uPAR+ extracellular vesicles as a cell-free therapy option may serve as one of the promising approaches for targeting key links in the pathogenesis of heart diseases.

Our findings that uPAR modulates signals promoting survival and proangiogenic properties of c-kit+ MPCs suggest that targeting this receptor is reasonable for overcoming the problems of poor survival and engraftment of transplanted cells, which could have significant prospects for the development of regenerative technologies for heart diseases.

## 4. Materials and Methods

### 4.1. Animals and Ethics Statement

We used 10–14-week male mice lacking the *Plaur* gene (uPAR−/− mice), originally derived from C57Bl6/SV129 mice by Flanders Institute for Biotechnology (Belgium) and wild-type mice (Wt) of matching age as a control [32], provided by the Faculty of Medicine of Lomonosov Moscow State University. Neonatal Wt mice (2 days old) were used to isolate cardiac endothelial cells for tube assay experiments. All animal experiments carried out for the study were conducted in accordance with the Code of Practice for the Housing and Care of Animals Used in Scientific Procedures, American Association for Laboratory Animal Science, and Institute of Experimental Cardiology guidelines. Animal procedures were approved by the Ethics Board of Institutional Animal Care and Use Committee of the National Medical Research Center of Cardiology Named after Academician E.I. Chazov (permit number 385.06.2009).

### 4.2. CD117+ MPCs Isolation

Adult CD117+ MPCs were isolated from 10–14 week-old Wt mice as previously described [33] with modification. Briefly, mice were euthanized by cervical dislocation. The heart was cannulated on a Langendorff apparatus and perfused with warmed basic buffer followed by digestion with collagenase II solution (Worthington, Columbus, OH, USA). The cell suspension was passed through a 40-micron filter and then used for Lineage cell depletion (Direct Lineage Cell Depletion Kit (Myltenyi biotec, Bergisch Gladbach, Germany) and subsequent CD117+ MPC selection (CD117-conjugated Miltenyi magnetic microbeads (Myltenyi biotec, Bergisch Gladbach, Germany). Cells were grown in MPCs growth medium, which consisted of DMEM/F12 (Gibco, Paisley, Scotland, UK), 10% fetal bovine serum (ATCC, Manassas, VA, USA), basic fibroblast growth factor (20 ng/mL), epidermal growth factor (20 ng/mL) (all from Peprotech, London, UK), leukemia inhibitory factor (10 ng/mL) (Sigma, St. Louis, MI, USA), insulin-transferrin-selenium (1% (*v*/*v*)) (Gibco, Paisley, Scotland, UK), penicillin-streptomycin (1% (*v*/*v*)) (Gibco, Paisley, Scotland, UK).

### 4.3. uPAR Downregulation in MPCs

uPAR was silenced in CD117+ MPCs by transducing these cells with a lentivirus that encodes shRNA targeting mouse uPAR (Santa Cruz, CA, USA). Lentivirus that encodes scrambled shRNA (Santa Cruz, CA, USA) was used as the control for experiments using targeted shRNA. Transduced cells were selected with 2 μg/mL puromycin for 8 days. uPAR protein expression was then assessed by immunoblot analysis.

### 4.4. Clonogenic Assay

Single cells were plated into each well of the fibronectin-coated plate (Imtek, Moscow, Russia) with 100 μL of MPC growth medium per well. On day 14, count the number of clones generated in each plate and calculate the percentage of single cells plated per plate that have generated clonal colonies.

### 4.5. MTT—Assay

Cell viability was tested using the methylthiazolyltetrazolium (MTT) assay as described previously [34] with modifications. MPCs were plated in 96-well culture plates (5 × 10^3^ cells/well) in DMEM/F12/1% FBS in a final volume of 0.1 mL. Cells were incubated for 24, 48, and 72 h, and then 20 µL of 3-(4.5-dimethylthiazol-2-yl)-2.5-diphenyltetrazolium bromide (stock 2.5 mg/mL) was added to the culture medium for another 4 h. Formazan crystals were solubilized by adding 0.1 N HCl in isopropanol. Absorbance was measured at 595 nm (reference filter 620 nm) using a Multiscan Microplate Reader (Labsystems, Markham, ON, Canada) and the number of cells per well was calculated using a plotted standard curve.

### 4.6. Caspase Assay

Apo-one1 Homogeneous Caspase-3/7 Assay kit (Promega, Madison, WI, USA) was used to measure the caspase activity in MPCs according to the manufacturer’s instructions. Briefly, approximately 10,000 uPAR(+) and uPAR(−) MPCs were plated per well. The next day, the medium was changed to serum-free media. After 3 days, the caspase activity in the cells was measured by adding the pro-fluorescent substrate and reading the fluorescence at Ex/Em 499/521 nm (Perkin Elmer, Waltham, MA, USA).

### 4.7. Western Blot Protocol

The cells were lysed in RIPA buffer (50 mM Tris–HCl, pH 7.4, 150 mM NaCl, 1 mM EDTA, 1% Triton X-100, 0.5% sodium deoxycholate and 0.1% SDS) supplemented with protease inhibitor cocktail (Sigma, St. Louis, MI, USA) and 1 mM phenylmethylsulfonyl fluoride (Sigma, St. Louis, MI, USA) and incubated on ice for 1 h. For experiments determining AKT signaling activation cells were serum-starved, incubated with uPA (10 nM) in serum-free medium, and lysed in RIPA buffer. After removing the cell debris by centrifugation at 14,000× rpm for 15 min, samples were separated by 8–10% SDS–PAGE under reducing conditions and electrotransferred to PVDF membrane (Millipore, Bedford, MA, USA). The membranes were blocked with 5% (*w*/*v*) non-fat dry milk in Tris-buffered saline (TBS) supplemented with 0.1% Tween 20 for 1 h at room temperature. Primary antibodies (anti-uPAR (Bio-Rad (Hercules, CA, USA)), -CD117 (LifeSpan BioSciences, Lynnwood, WA, USA), -AKT (Cell signaling (Boston, MA, USA)), -pAKT (Cell signaling (Boston, MA, USA)), and -Gapdh (Cell signaling (Boston, MA, USAUSA))) were applied and incubated at 4 °C overnight. The membranes were then washed and probed with secondary horseradish peroxidase(HRP)-conjugated polyclonal antibodies (Jackson immuneResearch, West Grove, PA, USA) for 1 h at room temperature and developed using an ECL Western Blotting kit (Amersham Biosciences, USA). All expression data were normalized to Gapdh. The blots were subjected to densitometric analysis using Image J software (National Institutes of Health, USA).

### 4.8. Flow Cytometry

To determine surface expression CD117 (Becton Dickenson (Franklin Lakes, NJ, USA)), mesenchymal stem cell (anti-CD105, -CD73, -CD90 antibodies from Becton Dickenson (Franklin Lakes, NJ, USA)) and hematopoietic (anti-CD45, -CD34 antibodies from Becton Dickenson (Franklin Lakes, NJ, USA)) cell markers MPCs were washed twice with ice-cold PBS, resuspended in ice-cold PBS supplemented with 1% BSA, and incubated with primary labeled or isotype control antibodies for 30 min at 4 °C. The stained cells were washed with ice-cold PBS and fixed in PBS containing 1% paraformaldehyde. For the analysis of uPAR, MPCs were incubated with primary unlabeled rat-anti mouse uPAR (R&D Systems (Minneapolis, MN, USA)) antibodies for 30 min at 4 °C, washed with ice-cold PBS, incubated with secondary anti-rat-Alexa 488 antibodies for 30 min at 4 °C, and washed with ice-cold PBS and fixed in PBS containing 1% paraformaldehyde. Flow cytometry analysis was performed with a FACS Aria III (Becton Dickinson, Franklin Lakes, NJ, USA).

### 4.9. Myocardial Infarction Model and Cell Transplantation

Myocardial infarction was induced in adult Wt mice (8 weeks old) as previously described [33]. Briefly, mice were anesthetized with avertin, intubated, and placed on a mechanical ventilator MiniVent (Hugo Sachs Elektronik Harvard Apparatus GmbH, March, Germany). For induction of myocardial infarction, ligation of the proximal portion of the left coronary artery was performed through an access in the 9th left intercostal space. In the sham operation group, a thoracotomy was performed to expose the heart, but no suture was made to the coronary artery.

After myocardial infarction, mice were intramyocardially injected with 50 μL of PBS containing 10^5^ uPAR(+) or uPAR(−) MPCs. Animals in the control group were injected with PBS without cells. The hearts were harvested (72 h for cell retention and 14 days—for angiogenesis analysis) for further studies after euthanasia.

### 4.10. Posttransplantstion MPCs Retention and Angiogenesis Analysis

Hearts were excised under deep anesthesia with inhalation of 5% isoflurane. Before the hearts were harvested, 0.1 mL saturated KCl had been injected into the left ventricular chamber to arrest them in diastole. The atriums and large vessels were resected and washed with normal saline, embedded in the Tissue-Tek OCT compound (Sakura, Tokyo, Japan), and frozen in liquid nitrogen. The hearts were stored at −70 °C and sliced (7 μm thickness) transversely from the apex to the base of the left ventricular. To assess the survival of MPCs CellTracker™ CM-DiI+ cells (ThermoFisher Scientific (Waltham, MA, USA)) after transplantation, cardiac slices were fixed in 3.7% paraformaldehyde and stained with DAPI followed by a manual count of the labelled cells.

To assess vascularization of the damaged area after infarction, cardiac slices were stained with antibodies to the endothelial marker CD31. Sections were fixed in ice-cold acetone for 20 min, air-dried, and washed in PBS. After washing, slides were blocked with 10% normal donkey serum for 30 min. Antibodies were diluted in blocking solution (1% BSA in PBS) and sections were incubated with rat anti-mouse CD31 antibody ((Becton Dickenson (Franklin Lakes, NJ, USA)). Then slides were washed in PBS and incubated in a mixture of goat anti-rat AlexaFluor594-conjugated antibodies (Life Technologies, Carlsbad, CA, USA) for 40 min. At the end of incubation, nuclei were stained with DAPI, and sections were mounted under coverslips. Microphotographs were taken under 200× magnification in random fields of view located in the peri-infarction area of the section. Vessel counts (total number of CD31+ vessels in periinfarction zones) were performed manually in Image J software (https://imagej.nih.gov/ij/download.html; accessed on 29 September 2023).

### 4.11. Immunohistochemical Analysis

Expression of CD117 (LifeSpan BioSciences (Lynnwood, WA, USA)), vascular markers (CD31 ((Becton Dickenson (Franklin Lakes, NJ, USA)), and smooth muscle actin (Abcam, (Boston, USA)) in the hearts of Wt and uPAR−/− mice was detected by immunohistochemical staining of tissue cryosections. The staining procedure was performed using ImmPRESS polymer reagents, ImmPACT DAB, and phosphatase substrates (Vector lab, Newark, CA, USA) according to the manufacturer’s recommendations. Vessel (CD31-positive structures without lumen and colocalization with SMA) and CD117+ cell counts were performed manually in Image J software.

### 4.12. MPCs Secretion Analysis

Samples of conditioned media of uPAR(+) and uPAR(−) MPCs were obtained by culturing in DMEM/F12 medium without supplements for 24 h. Secretome analysis was performed by Proteome Profiler Mouse Angiogenesis Array Kit (R&D Systems, Minneapolis, MN, USA) according to the manufacturer’s protocol.

### 4.13. Mouse Neonatal Cardiac Endothelial Cell Isolation and Tube Assay

Mouse neonatal cardiac endothelial cells were harvested by enzymatic homogenization of neonatal mouse hearts (2 days old) using a Neonatal Heart Dissociation Kit (Miltenyi Biotec, Gaithersburg, MD, USA) followed by immunomagnetic selection with a Neonatal Cardiac Endothelial Cell Isolation Kit (Miltenyi Biotec, USA). Conditioned mediums collected from uPAR(+) and uPAR(−) MPC cells were tested for induction of angiogenesis in mouse cardiac endothelial cells. The tube formation assay was performed as described previously [35] with modifications. Briefly, endothelial cells were plated on a 48-well plate coated with Matrigel^®^ (Corning) and treated with uPAR(+) and uPAR(−) MPCs condition mediums. Images of forming tubes were captured 17 h later. The total number and length of tube-like structures per field were measured using Image J software (National Institutes of Health, Bethesda, MD, USA).

### 4.14. Statistical Analysis

Values are presented as mean ± SD or ±SEM. Statsoft “Statistica 8.0” was used for data analysis. Statistically significant differences between the two groups were determined by a Mann–Whitney U-test depending on the sample distribution profile. Multiple groups were compared using ANOVA with Bonferroni correction where required. *p*-values less than 0.05 were considered indicative of significance.

## Data Availability

Not applicable.
